# Application and analysis of retroperitoneal laparoscopic partial nephrectomy with sequential segmental renal artery clamping for patients with multiple renal tumor: initial experience

**DOI:** 10.1186/s12894-017-0272-9

**Published:** 2017-09-11

**Authors:** Jundong Zhu, Fan Jiang, Pu Li, Pengfei Shao, Chao Liang, Aiming Xu, Chenkui Miao, Chao Qin, Zengjun Wang, Changjun Yin

**Affiliations:** Department of Urology, The First Affiliated Hospital of Nanjing Medical University, Nanjing, 300 Guangzhou Road, Nanjing, 210029 China

**Keywords:** Kidney neoplasms, Retroperitoneal laparoscopic operation, Partial nephrectomy, Segmental renal artery, Sequential clamping

## Abstract

**Background:**

To explore the feasibility and safety of retroperitoneal laparoscopic partial nephrectomy with sequential segmental renal artery clamping for the patients with multiple renal tumor of who have solitary kidney or contralateral kidney insufficiency.

**Methods:**

Nine patients who have undergone retroperitoneal laparoscopic partial nephrectomy with sequential segmental renal artery clamping between October 2010 and January 2017 were retrospectively analyzed. Clinical materials and parameters during and after the operation were summarized.

**Results:**

Nineteen tumors were resected in nine patients and the operations were all successful. The operation time ranged from 100 to 180 min (125 min); clamping time of segmental renal artery was 10 ~ 30 min (23 min); the amount of blood loss during the operation was 120 ~ 330 ml (190 ml); hospital stay after the operation is 3 ~ 6d (5d). There was no complication during the perioperative period, and the pathology diagnosis after the surgery showed that there were 13 renal clear cell carcinomas, two papillary carcinoma and four perivascular epithelioid cell tumors with negative margins from the 19 tumors. All patients were followed up for 3 ~ 60 months, and no local recurrence or metastasis was detected. At 3-month post-operation follow-up, the mean serum creatinine was 148.6 ± 28.1 μmol/L (*p* = 0.107), an increase of 3.0 μmol/L from preoperative baseline.

**Conclusions:**

For the patients with multiple renal tumors and solitary kidney or contralateral kidney insufficiency, retroperitoneal laparoscopic partial nephrectomy with sequential segmental renal artery clamping was feasible and safe, which minimized the warm ischemia injury to the kidney and preserved the renal function effectively.

## Background

Current studies found that of retroperitoneal laparoscopic partial nephrectomy can be a nephron-sparing option which have no significant difference with radical nephrectomy in the aspect of oncologic efficacy to properly selected patients [1] and this novel technique has gained popularity widely in the world [2–4]. In regard to some patients with solitary kidney or contralateral kidney insufficiency, partial nephrectomy can effectively reduce the incidence of dialysis on account of postoperative kidney failure [5]. In traditional partial nephrectomy, surgeons conduct the tumor excision and wound suture in the state of complete renal ischemia with main renal artery clamped,which is usually complete within 20 ~ 30 min to avoid the kidney from irreversible injury [6]. It’s extremely difficult to complete the surgery within a specified time when multiple renal carcinoma exist. In view of this, we retrospectively analyzed the clinical data of 9 patients from October 2010 to January 2017 who underwent retroperitoneal laparoscopic partial nephrectomy with sequential segmental renal artery clamping which avoided the complete renal ischemia during the whole surgical process. The curative effect is satisfied, report as follows.

## Methods

### Clinical data

**Fig. 1:**
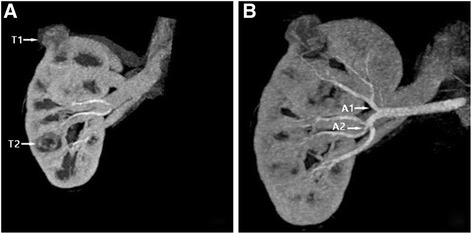
CTA was used to position tumors and their corresponding segmental arteries. **a** CTA showed the tumors in the upper (T1) and lower pole (T2) of right renal; **b** Superior, anterior and posterior branch were separated from the right renal artery near the renal hilum. Superior branch supplied the lesions in the upper pole (A1) and anterior branch supplied the lesions in the lower pole (A2)

From October 2010 to January 2017, a total of 756 partial nephrectomies were performed in our center and of these, a total of 9 patients including five male and four female underwent retroperitoneal laparoscopic partial nephrectomy with sequential segmental renal artery clamping, aging from 37 to 65 (mean of 51). Table 1 summarizes the demographic data for these patients. Six patients’ tumors were located in the left renal and three were located in the right renal. There were three cases of contralateral renal atrophy and six of solitary kidney. All the nine patients were diagnosed by physical examination with no obvious symptom of lumbago and hematuria. Eight of them had two tumors and one had three tumors in unilateral renal. Tumors were exophytic with the R.E.N.A.L score ranged from 4 to 6 points (mean of 4.4 points) and the diameter of them ranged from 1.8 to 3.5 cm (mean of 2.5 cm). No lymph nodes, renal vein or inferior vena cava tumor thrombus and distant metastasis were found in any patients. CT arteriography (CTA) was used preoperatively to show the tumors’ segmental arteries [7] (Figure 1). The first postoperative follow-up was performed at 1 month after operation with the examination of abdominal ultrasonography, chest radiography, hemogram, erythrocyte sedimentation rate (ESR) and blood biochemical tests. The follow-up of glomerular filtration rate (GFR), abdominal CT scan and hemogram were performed at 3th months postoperatively and reviewed every 3 months after that until 1 year. Afterwards, blood and imagine examinations were conducted annually.

**Table 1:** Characteristics of the recruited patients

Age	
Mean (range), year	51 (37–65)
Gender	
Male (%)	5 (56)
Female (%)	4 (44)
Tumor side	
Right (%)	3 (33)
Left (%)	6 (67)
Number of tumor	
Two tumors (%)	8 (89)
Three tumors (%)	1 (11)
Tumor size	
Mean (range), cm	2.5 (1.8–3.5)
R.E.N.A.L score	
Mean (range)	4.4 (4–6)

### Surgical methods

**Fig. 2:**
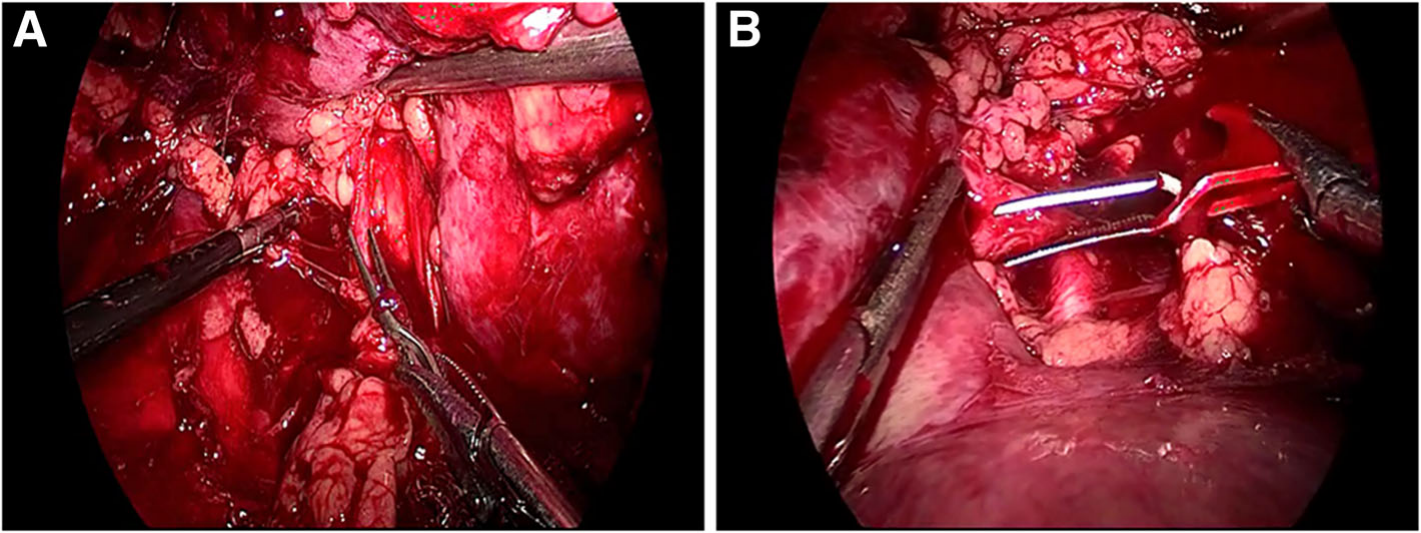
Clamping the superior and anterior branch of the right renal artery during the operation. **a** clamping the superior branch before resecting the tumor in upper pole; **b** clamping the anterior branch before resecting the tumor in lower pole

Patients were administered general anesthesia and placed in the lateral decubitus position. To establish the retroperitoneal space, the trocar was inserted according to the location of the tumors. Then, 2 cm incision was made via the median axillary line at the level of iliac crest. A self-made balloon was placed in the retroperitoneal space through the incision and infused 800 ml air to expand the retroperitoneal space. Under the guidance of fingers, a 12 mm trocar was inserted below the 12th rib at the posterior axillary line. Two 5 mm trocars were placed up and down at the anterior axillary line and observation mirror was inserted through the incision above the illac crest. Pneumoperitoneum pressure was maintained at 15 mmHg (1 mmHg = 0.133kpa). In the case of patients with ventral tumors needing to perform the segmental renal artery clamping in the front of renal hilar, all trocar would be moved toward anterior median line by 2 ~ 3 cm [8]. The fascia and adipose capsule of kidney were opened along the dorsal renal to make the kidney dissociative, revealing renal tumor and peripheral renal parenchyma. Instead of separating the renal artery trunk, we dissociated several segmental arteries of the tumor directly in the vicinity of the renal hilar under the guidance of the CTA (Fig. 2). Subsequently, the segmental artery was clamped and a scissor was used to resect the tumor in the renal parenchyma, 2 mm away from the tumor edge. After the wound was continuous sutured with absorbable suture, releasing the segmental arteries to check if any active bleeding existed. In the same manner, clamping the other segmental arteries sequentially to resect other tumors. After resecting all the renal tumors, renal blood flow was restored and no obvious hemorrhage should be confirmed again at the place of wound, renal hilar and every puncture hole. Finally, packing the resected tumors into the specimen bag to fetch them out, then indwelling the drainage tube and closing the incision.

### Statistical analysis

Statistical analysis of pre- and postoperative serum creatinine (SCr) levels were performed using the student t test. All data are reported as mean and range, with *p* < 0.05 considered statistically significant.

## Results

All operations were successfully completed and 19 tumors were cut off from nine patients. Operative characteristics are presented in Table 2. The operation time ranged from 100 to 180 min (125 min) and warm ischemia time of each 19 clamping was 10 ~ 30 min (23 min). The blood loss during operations was 120 ~ 330 ml (190 ml) and the postoperation hospital stays was 3 ~ 6 days (5 days). There was no complication in perioperative period. After the surgery, we routinely conducted a clinicopathologic analysis and the results showed 13 renal clear cell carcinomas, two papillary carcinoma and four perivascular epithelioid cell tumors with negative margins from the 19 tumors. All patients had no local recurrence or metastasis with a 3 ~ 60 months follow-up visit (16.6 months).

**Table 2:** Operative characteristics of the operation

Operation time	
Mean (range), min	125 (100–180)
Warm ischemia time of each tumor	
Mean (range), min	23 (10–30)
Blood loss	
Mean (range), ml	190 (120–330)
Postoperation hospital stays	
Mean (range), day	5 (3–6)
Histology	
Clear cell carcinoma (%)	13 (68)
Papillary carcinoma (%)	2 (11)
Perivascular epithelioid cell tumors (%)	4 (21)
Postoperative follow-up time	
Mean (range), month	16.6 (3–60)

Mean preoperative SCr was 145.6 ± 25.9 μmol/L respectively and the mean SCr increased to 182.1 ± 26.6 μmol/L (*p* < 0.001) at 1 day and 197.4 ± 34.2 μmol/L (p < 0.001) at 3 day after the operation. Mean SCr at 1-month follow-up showed dramatic recoveries and reached to 156.7 ± 28.4 μmol/L (*p* = 0.002). At 3-month post-operation follow-up, the mean SCr was 148.6 ± 28.1 μmol/L (*p* = 0.107), only an increase of 3.0 μmol/L from baseline (Table 3).

**Table 3:** Pre-operative and post-operative comparisons of serum creatinine

	Pre-Operation	Post-Operation at 1 day	Post-Operation at 3 day	Post-Operation at 1 month	Post-Operation at 3 month
mean(μmol/L)	145.6 ± 25.9	182.1 ± 26.6	197.4 ± 34.2	156.7 ± 28.4	148.6 ± 28.1
*P*-value		<0.001	<0.001	0.002	0.107

## Discussion

Shortening the warm ischemia (WI) time and reducing the area of WI are the main methods to protect renal function in partial nephrectomy [9]. Nowadays, continuous main renal artery clamping is the main method to block the bloodstream in partial nephrectomy and studies show that the renal function will be damaged irreversibly if exceeding the renal tolerant time of WI for 30 min.Therefore, surgeons were supposed to complete the resection and suture within 30 min after clamping renal artery in partial nephrectomy. To reduce the area of WI, unblocking and segmental renal artery clamping technique was applied. Although the operation demand high equipment quality and operation technique, more and more departments carrying out these techniques in recent years [10–12]. What’s more, our previous study has been demonstrated that patients underwent segmental artery clamping have better early recovery comparing by 3-month postoperative GFR levels [13].

In clinical practice, it is usually difficult to handle multiple renal tumors with solitary kidney and contralateral kidney insufficiency. Patients are supposed to receive postoperative dialysis treatment if underwent a radical nephrectomy. Because of multiple lesions, nephron-sparing operation is hardly to be completed within 30 min to protect renal away from irreversible damage. Therefore, some scholars have proposed a scheme to protect renal function by reducing renal temperature [14]. In addition, under the pressure of limited time for surgeons, the probability to rupture the tumor capsule, suture the wound and collection system imprecisely during the operation will increase and thus affect the prognosis and complications rates. For these special patients, we complete the partial nephrectomy by applying the technique of retroperitoneal laparoscopic partial nephrectomy with sequential segmental renal artery clamping and protecte the patient’s renal function satisfyingly by reducing the area of WI during the operation. The advantages of this technique are as follows: ① Complete renal ischemia can be avoided during the operation and the blood supply of other parts of kidney is unaffected when segmental renal arteries are clamped, so that the renal function can be protected effectively. ②surgeons can complete each resection and wound suture carefully instead of dealing with several tumors in a hurry time, reducing surgical complications and ensuring the operation efficacy.

We believe that the key points of this technique are as follows:

① Before the operation, doctor should establish a three-dimensional renal artery reconstruction model by imagine techniques to determine the number and block position of target vessels, and design the path to separate the target vessels [7]. ② Position of the trocar should be adjusted according to the surgical approach which will make the the target vessels and tumors separated easily [8]. ③ After recognizing the anatomic spatial relationships according to the three-dimensional renal artery reconstruction model, surgeons can directly attempt to free the segmental artery in the renal hilum by a scissor instead of the equipment with heating effect such as coagulation hook and harmonic scalpel [13]. ④ Providing tumors are resected completely, interlobular vessels outside the tumor psuedocapsule should be reserved as much as possible in order to maximally save the blood supply of peripheral normal renal tissue.

Surgeons should adequately evaluate tumor location, size, exophytic or not and the complexity of feeding artery to each kidney segment if they decide to conduct sequential segmental renal artery clamping for patients with multiple renal tumor. If the tumor is too close to the hilum, it is hard to achieve clear exposure of segmental arteries and pelvis without compression of the tumor, so conventional method is more suitable for these cases. What’s more, surgical method should also be considered cautiously for the patients with bigger or overmany tumors or already metastasize to peripheral renal tissue or lymph nodes. In our selected patients, the average diameter of these tumors is 2.5 cm and all of them were exophytic. Therefore, the surgical procedure was not difficult and the local WI time of each clamping can be controlled within 30 min during the operation. Due to sufficient preoperative assessment, no cases converted to main renal artery clamping or open operation in our study. No lymph nodes, renal vein or inferior vena cava thrombus and distant metastasis were found among the nine patients, so that the renal function and oncological efficacy is better with no subsequent dialysis.

Nonetheless, limitations of this study exist regarding the sample size and retrospective nature of the study. Due to the case number constraints of multiple renal tumors with solitary kidney or contralateral kidney insufficiency, this study does not compare sequential selective clamping to main renal artery clamping for multiple tumor removal; rather, it presents a feasible technique for minimizing the warm ischemia injury to the kidney and preserving the renal function.

## Conclusions

In conclusion, retroperitoneal laparoscopic partial nephrectomy with sequential segmental renal artery clamping can avoid complete renal WI injury and the operation is safe and feasible. This technique can be selectively applied according to the particular tumor situation for the patients with isolated kidney, contralateral renal insufficiency or bilateral multiple renal tumors.

## Abbreviations

CT: Computed tomography
CTA: Computed tomography arteriography
ESR: Erythrocyte sedimentation rate
GFR: Glomerular filtration rate
SCr: Serum creatinine
WI: Warm ischemia
